# Data on drinking water quality using water quality index (WQI) and assessment of groundwater quality for irrigation purposes in Qorveh&Dehgolan, Kurdistan, Iran

**DOI:** 10.1016/j.dib.2018.08.022

**Published:** 2018-08-13

**Authors:** Hamed Soleimani, Omid Nasri, Boshra Ojaghi, Hasan Pasalari, Mona Hosseini, Bayram Hashemzadeh, Ali Kavosi, Safdar Masoumi, Majid Radfard, Amir Adibzadeh, Ghasem Kiani Feizabadi

**Affiliations:** a Department of Environmental Health Engineering, School of Public Health, Tehran University of Medical Sciences, Tehran, Iran; b Department of Environmental Health, School of Public Health, Iran University of Medical Sciences, Tehran, Iran; c Department of Environmental Health Engineering, School of Health, Isfahan University of Medical Sciences, Isfahan, Iran; d Khoy University of Medical Sciences, Khoy, Iran; e Nursing Research Center, Faculty Member Golestan University of Medical Sciences, Gorgan, Iran; f Department of Epidemiology and Biostatistics, School of Public Health, Tehran University of Medical Sciences, Tehran, Iran; g Health Research Center, Life Style Institute, Baqiyatallah University of Medical Sciences, Tehran, Iran; h Department of Environmental Health Engineering, School of Health, Semnan University of Medical Sciences, Semnan, Iran

**Keywords:** Groundwater, WQI, Irrigation, Kurdistan, Iran

## Abstract

This data article aimed to investigate the quality of drinking water of Qorveh and Dehgolan Counties in Kurdistan province based on the water quality index (WQI) and agricultural quality index based on RSC, PI, KR, MH, Na, SAR and SSP indices. Also, Piper diagram was used to determine hydro chemical features of the groundwater area. The calculation of WQI for groundwater samples indicated that 36% of the samples could be considered as excellent water and 64% of the samples were classified as good water category. The results of the calculated indices for agricultural water quality indicate that water quality in all collected samples are in a good and excellent category. The Piper classification showed that dominant type of groundwater hydro chemical faces of region was calcium bicarbonate (Ca-HCO_3_^−^).

**Specifications Table**

**Table:** 

Subject area	Chemistry
More specific subject area	Water quality
Type of data	Tables, Figures
How data was acquired	All water samples were analyzed according to the Standard Methods for Examination of Water and Wastewater and using titration method permanent hardness, magnesium and calcium were measured.
Data format	Raw, Analyzed
Experimental factors	All water samples in polyethylene bottles were stored in a dark place at room temperature until the metals analysis
Experimental features	The mentioned parameters above, in abstract section, were analyzed according to the standards for water and wastewater.
Data source location	Qorveh&Dehgolan, Kurdistan province, Iran
Data accessibility	Data are included in this article

**Value of data**

• Based on limited surveys in Qorveh-Dehgolan, the data can contribute to an understanding of the quality of groundwater in the region and to provide further studies on the quality of water for drinking and agriculture purposes.
• The water quality indexes (WQI) show useful information on the quality of drinking water. Therefore, these data could be useful for communities or cities that have similar drinking water quality.
• The data of the calculated water quality index (WQI) can be helpful for irrigation purposes.
• Piper diagram can be used to determine hydro chemical features of the groundwater.

## 1 Data

**Fig. 1:**
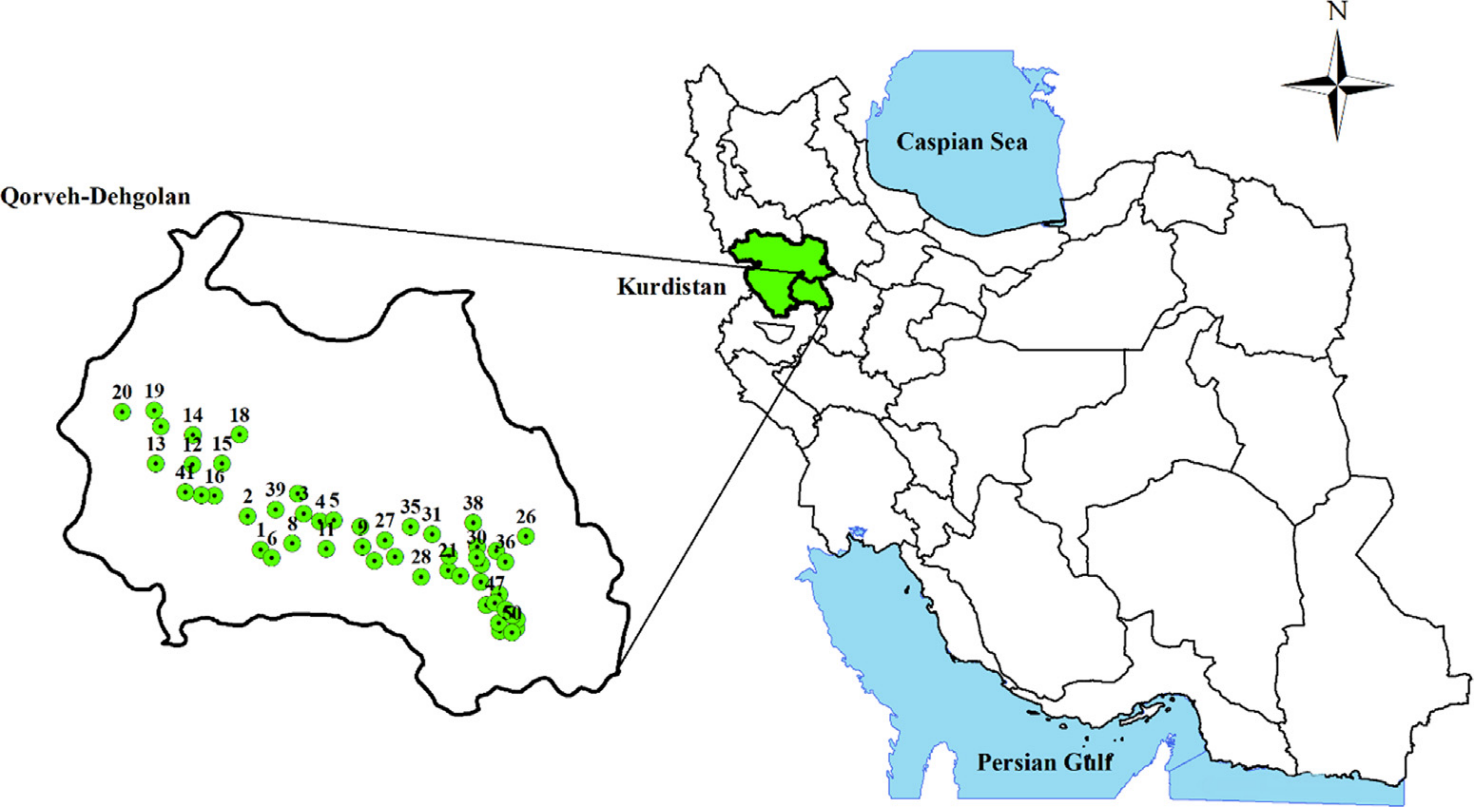
Location of the study area.

**Fig. 3:**
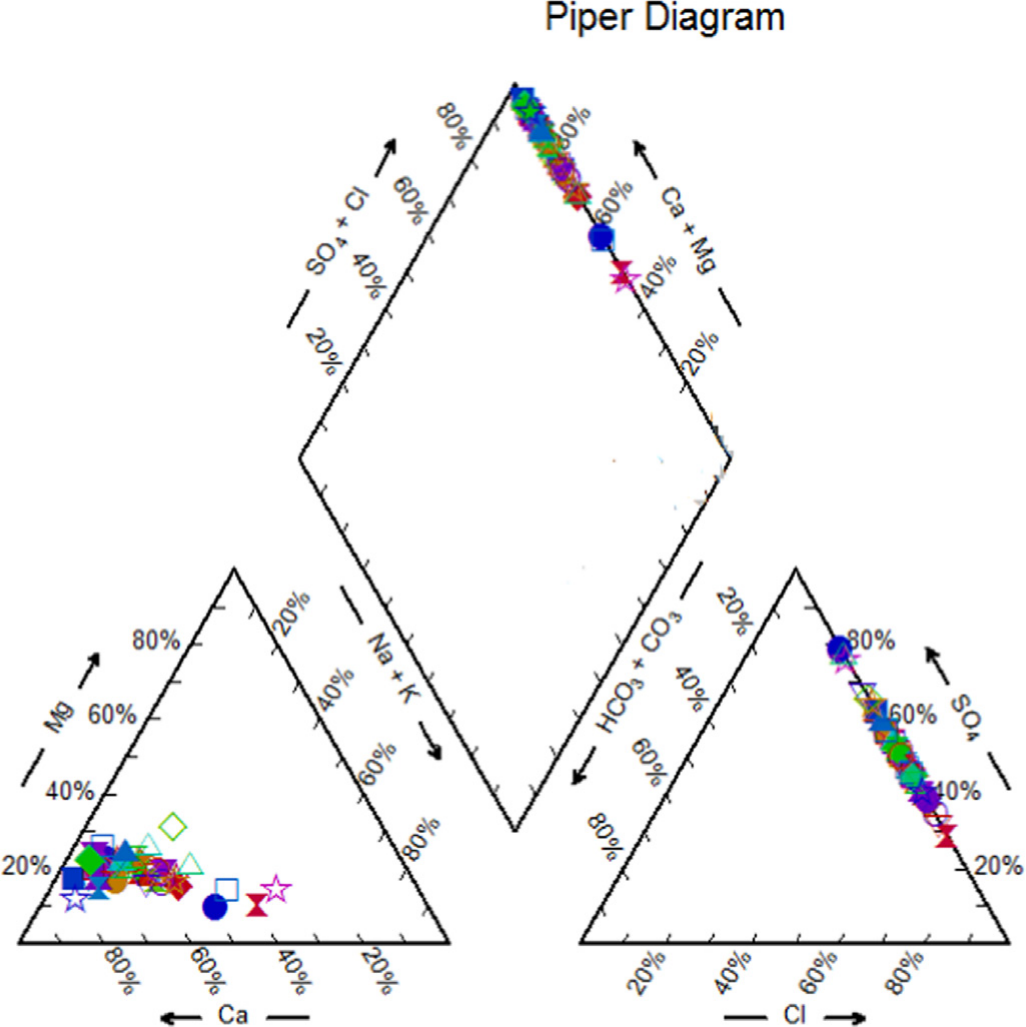
Piper diagram of groundwater samples of the present study.

Concentration of studied physicochemical parameters in the groundwater of Iran, Kurdistan province, and water sampling situations are summarized in Table 1 and Fig. 1. Based on the data of the WQI index calculation, water quality can be classified into five classes, as shown in Table 1, Table 2, Table 3. Also, the classification of groundwater samples for use of irrigation in EC, SAR, RSC, KR, SSP, PI, MH, Na%, TH and, as well as The calculated results are presented for these indices in Table 5, Table 6, Table 7, respectively. To obtain the correlation of scale variables we used Spearman correlation coefficient, which is shown in Table 8. Finally, the Piper diagram shows that the hydro chemical type of water is Ca-HCO_3_^−^ (Fig. 3) and also, water quality index (WQI) classification for individual samples has been shown in (Table 4).

**Table 1:** Physico-chemical and statistically analyzed water quality parameters.

Well number	Type of water source	UTM			pH	EC	TDS	TH	Ca^2+^	Mg^2+^	Na^+^	K^+^	SO_4_^2^^−^	HCO_3_^−^	Cl^−^
		Y		X		μmhos/cm	mg/L	mg/L	mg/L	mg/L	mg/L	mg/L	mg/L	mg/L	mg/L
W1	Deep well	35.168068	47.498878		7.85	480	307	228	69	13.431	15.18	0.39	11.04	273.28	8.165
W2	Semi-deep well	35.219263	47.472425		8.08	330	211	138	42	7.986	18.86	0.39	3.84	196.42	3.195
W3	Deep well	35.226098	47.577664		8.02	370	237	166	50	9.922	15.18	0.39	4.8	216.55	4.615
W4	Deep well	35.214918	47.608515		7.9	430	275	210	65	11.495	10.12	0.78	5.76	246.44	7.1
W5	Deep well	35.217779	47.63454		7.78	494	316	172	54	8.954	43.01	0.78	39.84	241.56	10.295
W6	Deep well	35.156122	47.520717		7.73	526	337	260	78	15.73	12.19	0.39	11.04	298.9	9.585
W7	Semi-deep well	35.20883	47.685694		8.01	462	296	224	66	14.278	6.21	0.39	12	209.84	12.78
W8	Deep well	35.17943	47.557788		7.88	393	252	174	54	9.438	20.01	0.39	10.08	237.9	6.035
W9	Deep well	35.177842	47.690503		8.02	389	249	166	51	9.317	20.47	0.78	11.04	223.26	7.1
W10	Semi-deep well	35.256976	47.565125		7.7	454	291	230	74	10.89	8.74	0.39	10.08	272.67	5.325
W11	Deep well	35.172975	47.622313		8	415	266	186	56	11.132	17.94	0.78	10.08	235.46	8.52
W12	Semi-deep well	35.295814	47.365098		8	395	253	202	66	8.954	3.91	0.39	10.08	229.36	4.615
W13	Deep well	35.295077	47.29673		7.9	410	262	190	61	9.075	14.49	0.39	11.04	231.8	6.39
W14	Deep well	35.341614	47.364265		7.86	483	309	246	78	12.342	10.12	0.39	5.76	286.7	6.035
W15	Deep well	35.298951	47.420618		7.9	272	171	132	41	7.139	5.29	0.39	3.84	152.5	3.55
W16	Semi-deep well	35.248597	47.408867		8.1	449	287	116	37	5.687	54.05	0.39	11.04	222.04	20.59
W17	Deep well	35.352906	47.303318		7.96	461	295	192	60	10.164	27.14	0.78	11.04	268.4	8.165
W18	Deep well	35.344516	47.452034		8.15	311	199	140	41	9.075	11.5	0.78	10.08	170.8	5.68
W19	Deep well	35.376907	47.289813		7.91	650	416	292	71	27.709	34.04	1.56	39.84	353.8	15.62
W20	Deep well	35.373085	47.229557		8.1	450	288	218	67	12.221	10.12	0.78	10.08	234.85	9.585
W21	Deep well	35.14605	47.85312		8.02	320	205	146	43	9.317	13.8	0.39	4.8	195.2	4.615
W22	Deep well	35.137667	47.876034		8.55	314	201	130	38	8.47	18.17	0.39	5.76	140.3	5.325
W23	Deep well	35.157183	47.914739		7.9	326	209	154	48	8.228	6.9	0.39	3.84	169.58	4.615
W24	Deep well	35.168433	47.853804		7.75	524	335	158	53	6.171	49.91	0.78	44.16	247.66	8.875
W25	Deep well	35.164491	47.751927		7.88	410	262	204	64	10.648	8.05	0.39	10.08	228.75	7.1
W26	Deep well	35.201912	47.997529		7.91	447	286	170	53	9.075	32.89	0.78	14.88	248.88	12.07
W27	Deep well	35.189449	47.732478		7.8	382	244	154	48	8.228	23.92	0.39	4.8	216.55	6.745
W28	Deep well	35.134725	47.801978		7.8	438	280	186	57	10.527	23	0.78	12	251.32	5.325
W29	Deep well	35.183581	47.906559		7.7	619	396	286	92	13.552	14.95	0.78	18.24	258.64	28.4
W30	Deep well	35.167667	47.905684		7.9	374	239	180	54	10.89	11.27	0.39	4.8	219.6	4.26
W31	Semi-deep well	35.20112	47.820928		7.9	360	230	168	55	7.381	8.97	0.39	4.8	192.15	5.325
W32	Semi-deep well	35.156808	47.714154		7.75	622	398	240	73	13.915	43.01	1.17	54.72	273.28	32.305
W33	Deep well	35.111437	47.95028		7.8	390	250	186	62	7.502	10.58	0.78	7.68	221.43	5.325
W34	Deep well	35.178168	47.941868		7.83	375	240	190	58	10.89	4.83	0.39	5.76	211.06	5.325
W35	Deep well	35.211534	47.779489		8.1	362	232	166	50	9.922	11.5	0.78	10.08	192.76	6.39
W36	Deep well	35.161974	47.95947		8.1	330	211	136	41	8.107	19.55	1.17	10.08	172.63	7.455
W37	Semi-deep well	35.129474	47.914836		7.95	422	270	184	51	13.673	17.48	1.17	12	213.5	11.005
W38	Deep well	35.22061	47.897434		8.1	431	276	200	58	13.31	17.25	0.78	11.04	256.2	7.455
W39	Deep well	35.230987	47.524515		8	342	219	170	49	11.495	5.29	0.39	4.8	186.05	4.26
W40	Deep well	35.248876	47.384327		8.15	340	218	144	44	8.228	10.58	0.78	5.76	159.82	5.68
W41	Deep well	35.252743	47.353516		8.05	382	244	152	48	7.744	19.09	0.78	39.84	140.3	14.2
W42	Deep well	35.054453	47.953708		8	514	329	244	74	14.278	15.41	0.78	13.92	283.04	10.65
W43	Deep well	35.067023	47.951234		7.95	434	278	218	67	12.221	6.44	0.78	12	237.9	8.165
W44	Deep well	35.094387	47.926746		8	453	290	218	63	14.641	14.49	0.78	15.84	263.52	8.165
W45	Deep well	35.060411	47.98421		8	325	208	132.5	40.4	7.623	23	1.17	10.08	195.2	4.26
W46	Deep well	35.073174	47.984565		8.2	524	335	264	82	14.278	8.74	0.78	10.08	279.38	11.005
W47	Deep well	35.097664	47.94214		8	396	253	164	49	10.043	22.08	0.78	11.04	212.28	8.165
W48	Deep well	35.088025	47.962661		8.1	776	504	228	69	13.431	74.06	1.17	123.84	185.44	69.225
W49	Deep well	35.079689	47.975834		8	713	463	180	50	13.31	89.93	3.51	84.96	285.48	20.235
W50	Deep well	35.052643	47.975729		8	613	392	226	64	15.972	43.01	2.73	87.84	212.28	19.525
Mean					7.96	437.64	280.28	189.21	57.57	10.96	20.53	0.76	18.04	227.05	10.29
Max					8.55	776.00	504.00	292.00	92.00	27.71	89.93	3.51	123.84	353.80	69.23
Min					7.70	272.00	171.00	116.00	37.00	5.69	3.91	0.39	3.84	140.30	3.20
SD					0.15	106.26	68.92	41.94	12.53	3.54	17.41	0.57	23.81	43.48	10.41

**Table 2:** The weight (*w*_*i*_) and relative weight (*W_i_*) of each chemical parameter calculated based on the standard values reported by the World Health Organization [1], [2], [3].

Parameter	WHO guideline (mg/L)	Weight (*w_i_*)	Relative weights (*W_i_*)
[K^+^]	12	2	0.056
[Na^+^]	200	4	0.111
[Mg^+^]	50	3	0.083
[Ca^2+^]	75	3	0.083
[HCO_3_]	120	1	0.028
[Cl^−^]	250	5	0.139
[SO_4_]	250	5	0.139
[pH]	8.5	3	0.083
[TDS]	500	5	0.139
		Σ	Σ

**Table 3:** Water quality classification ranges and types of water based on WQI values [1], [4], [5], [6].

**Range**	**Type of groundwater**
< 50	Excellent water
50–99.99	Good water
100–199.99	Poor Water
200–299.99	Very poor water
≥ 300	Unsuitable for drinking/Irrigation purpose

**Table 4:** Water quality index (WQI) classification for individual samples.

Well number	DWQI	Water quality rating
W1	61.07	Good
W2	43.43	Excellent
W3	48.57	Excellent
W4	56.80	Good
W5	55.46	Good
W6	66.89	Good
W7	59.70	Good
W8	50.83	Good
W9	49.89	Excellent
W10	60.24	Good
W11	53.46	Good
W12	54.54	Good
W13	53.51	Good
W14	63.56	Good
W15	39.95	Excellent
W16	46.04	Excellent
W17	56.13	Good
W18	43.36	Excellent
W19	77.52	Good
W20	59.02	Good
W21	44.13	Excellent
W22	42.34	Excellent
W23	44.90	Excellent
W24	54.58	Good
W25	55.22	Good
W26	52.89	Good
W27	47.29	Excellent
W28	53.97	Good
W29	74.58	Good
W30	50.40	Good
W31	48.23	Excellent
W32	70.75	Good
W33	52.13	Good
W34	51.61	Good
W35	48.66	Excellent
W36	43.84	Excellent
W37	53.27	Good
W38	56.07	Good
W39	47.72	Excellent
W40	44.47	Excellent
W41	48.65	Excellent
W42	65.02	Good
W43	58.25	Good
W44	59.28	Good
W45	43.20	Excellent
W46	68.19	Good
W47	49.73	Excellent
W48	78.90	Good
W49	68.04	Good
W50	69.54	Good

**Table 5:** Summary of water quality indices in present study [7], [8], [9], [10], [11], [12], [13].

**Indices**	**Formula**
Residual sodium carbonate (RSC)	RSC = (CO_3_^2−^+HCO_3_^−^)+(Ca^2+^+Mg^2+^)
Permeability index (PI)	$\mathrm{PI}=\frac{\mathrm{Na}+K+\sqrt{\mathrm{HCO}3}}{\mathrm{Ca}+\mathrm{Mg}+\mathrm{Na}+K}\times 100$
Kelly’s ratio (KR)	$\mathrm{KR}=\frac{\mathrm{Na}}{\mathrm{Ca}+\mathrm{Mg}}$
Magnesium hazard(MH)	$\mathrm{MH}=\frac{\mathrm{Mg}}{\mathrm{Ca}+\mathrm{Mg}}\times 100\;$
Sodium percentage (Na %)	$\mathrm{Na}\%=\frac{\mathrm{Na}+K}{\mathrm{Ca}+\mathrm{Mg}+\mathrm{Na}+K}\times 100$
Sodium adsorption ratio (SAR)	$\mathrm{SAR}=\frac{\mathrm{Na}}{\sqrt{(\mathrm{Ca}+\mathrm{Mg})/2}}\times 100$
Soluble sodium percentage (SSP)	$\mathrm{SSP}=\frac{\mathrm{Na}}{\mathrm{Ca}+\mathrm{Mg}+\mathrm{Na}}\times 100\;$

**Table 6:** Calculation of RSC, PI, KR, MH, Na%, SAR and SSP of groundwater.

Well number	RSC	PI	KR	MH	Na%	SAR	SSP
w1	− 0.1	52.67608	0.140693	25.32468	12.5	0.427669	12.33397
w2	− 4.3	30.43871	0.095517	19.59064	8.880995	0.432681	8.718861
w3	0.26	64.87732	0.208589	23.31288	17.46835	0.532617	17.25888
w4	− 0.3	52.28866	0.103865	22.70531	9.606987	0.298871	9.40919
w5	− 0.22	53.89116	0.126829	19.5122	11.44708	0.363184	11.25541
w6	− 0.28	47.56944	0.103448	27.20307	9.532062	0.334252	9.375
w7	− 1	44.05674	0.057269	28.4141	5.613306	0.172568	5.416667
w8	0.46	66.4299	0.263158	22.51462	21.01617	0.688247	20.83333
w9	0.32	65.4821	0.260479	23.65269	21.04019	0.673226	20.66508
w10	− 0.14	50.14377	0.082969	19.21397	7.847082	0.251111	7.66129
w11	0.18	61.05971	0.208556	23.79679	17.62115	0.570392	17.25664
w12	− 0.34	49.39279	0.041463	19.5122	4.205607	0.118733	3.981265
w13	0.38	71.50413	0.534286	22.85714	35.06494	1.413587	34.82309
w14	− 0.34	52.14724	0.126728	22.81106	11.42857	0.373364	11.24744
w15	− 0.18	60.77683	0.108844	20.06803	10.36585	0.263932	9.815951
w16	1.39	91.91092	1.066964	21.875	51.72414	2.258338	51.61987
w17	0.5	64.45005	0.3	23.07692	23.37917	0.837854	23.07692
w18	0.19	65.51562	0.191489	29.07801	16.56805	0.454762	16.07143
w19	0.32	68.52513	0.212766	27.30496	17.78426	0.505291	17.54386
w20	− 0.51	48.93188	0.09589	22.37443	9.128631	0.28381	8.75
w21	0.14	52.71506	0.286787	30.93093	22.91667	1.046674	22.28705
w22	0.41	76.95	0.480315	31.10236	32.62599	1.082575	32.44681
w23	− 0.3	57.58688	0.092357	25.15924	8.72093	0.231445	8.45481
w24	0.78	74.75866	0.62	24.28571	38.48858	1.640366	38.2716
w25	− 0.07	58.96209	0.094771	23.20261	8.928571	0.234451	8.656716
w26	0.62	71.06918	0.411243	23.07692	29.43633	1.069231	29.14046
w27	0.02	61.54112	0.190058	22.51462	16.17647	0.497067	15.97052
w28	− 0.3	53.45452	0.174009	28.4141	14.98127	0.524341	14.82176
w29	− 1.86	40.33636	0.129738	20.55394	11.71171	0.480555	11.48387
w30	0.18	61.19696	0.156069	24.85549	13.71571	0.410554	13.5
w31	− 0.18	57.48031	0.114035	19.59064	10.4712	0.29824	10.23622
w32	− 0.58	56.58115	0.346304	27.0428	26.04317	1.110333	25.72254
w33	− 0.02	57.06826	0.121622	20.27027	11.05769	0.330847	10.84337
w34	− 0.34	51.38	0.052356	24.08377	5.210918	0.144715	4.975124
w35	− 0.03	59.85215	0.157576	25.75758	14.0625	0.404819	13.61257
w36	0.44	77.45903	0.418699	20.73171	30.31161	0.92872	29.51289
w37	− 0.18	58.25647	0.193122	31.21693	16.55629	0.530997	16.18625
w38	− 0.42	52.36534	0.117073	26.82927	10.86957	0.335247	10.48035
w39	− 0.32	54.00683	0.073099	26.90058	7.065217	0.19118	6.811989
w40	− 0.15	61.79381	0.162069	27.58621	14.45428	0.390314	13.94659
w41	− 0.7	55.80651	0.176301	26.30058	15.40342	0.463774	14.98771
w42	− 0.46	49.87868	0.127615	24.68619	11.6451	0.394576	11.31725
w43	− 0.34	49.66664	0.06338	24.88263	6.373626	0.185001	5.960265
w44	0.38	60.41969	0.201005	25.8794	17.08333	0.567105	16.7364
w45	0.66	80.72443	0.42562	23.55372	30.45977	0.936364	29.85507
w46	0.7	78.02725	0.773006	32.51534	43.7931	1.973816	43.59862
w47	0.13	65.07177	0.282209	24.84663	22.38095	0.7206	22.00957
w48	− 1.34	58.65971	0.503861	24.71042	33.75959	1.621775	33.50449
w49	1.22	80.83319	1.068306	15.30055	52.21932	2.890355	51.65125
w50	0.82	79.43416	0.914201	27.51479	48.2389	2.376923	47.75889

**Table 7:** Classification of groundwater sample for irrigation use on the basic of EC, SAR, RSC, KR, SSP, PI, MH, Na%, T.H.

**Parameters**	**Range**	**Water class**	**Samples (%)**
**EC**	< 250	Excellent	0
	250–750	Good	98
	750–2250	Permissible	2
	> 2250	Doubtful	0
**SAR**	0–10	Excellent	100
	10–18	Good	0
	18–26	Doubtful	0
	> 26	Unsuitable	0
**RSC**	< 1.25	Good	98
	1.25–2.5	Doubtful	2
	> 2.5	Unsuitable	0
**KR**	< 1	Suitable	96
	1–2	Marginal suitable	4
	> 2	Unsuitable	0
**SSP**	< 50	Good	96
	> 50	Unsuitable	4
**PI**	> 75	Class-I	8
	25–75	Class-II	92
	< 25	Class-III	0
**MH**	< 50	Suitable	100
	> 50	Harmful and Unsuitable	0
**Na%**	< 20	Excellent	60
	20–40	Good	32
	40–60	Permissible	8
	60–80	Doubtful	0
	>80	Unsuitable	0
**T.H**	< 75	Soft	0
	75–150	Moderately hard	18
	150–300	Hard	82
	> 300	Very hard	0

**Table 8:** Pearson’s correlation coefficient.

	pH	Na	K	Ca	Mg	SO	Cl	TDS	EC	HCO_3_	TH
pH	1										
Na	0.008	1									
K	0.077	0.681**	1								
Ca	− 0.437**	− 0.097	0.032	1							
Mg	− 0.102	0.11	0.383**	0.615**	1						
SO_4_	− 0.013	0.82**	0.71**	0.182	0.325*	1					
Cl	0.004	0.658**	0.373**	0.328*	0.308*	0.816**	1				
TDS	− 0.241	0.69**	0.594**	0.629**	0.629**	0.798**	0.774**	1			
EC	− 0.247	0.685**	0.591**	0.635**	0.634**	0.793**	0.77**	1	1		
HCO_3_	− 0.473**	0.198	0.217	0.698**	0.66**	0.118	0.095	0.619**	0.625**	1	
TH	− 0.362**	− 0.034	0.157	0.961**	0.808**	0.25	0.353*	0.69**	0.696**	0.752**	1

** Correlation is significant at the 0.01 level (2-tailed).

* Correlation is significant at the 0.05 level (2-tailed)

## 2 Experimental design, materials and methods

### 2.1 Study area

Our study area includes two counties: Qorveh county, and Dehgolan county. Qorveh and Dehgolan counties in Kurdistan province are located in west of Iran. Qorveh is located between the latitudes 35.1679°N and longitudes 47.8038°E, encompassing an area of about 4338.7 km^2^ and the average altitude of the city is 1900 m above sea level. Dehgolan is located between the latitudes 35.2798 °N and longitudes 47.4221°E. also. The area of this county is 2050 km^2^ and the average altitude of the city is 1800 m above sea level.

### 2.2 Sample collection and analytical procedures

For the purpose of this data article, a total of 50 rural drinking water sources were collected in Qorveh-Dehgolan area in Kurdistan province, for 12 months (2015–2016). Water samples were analyzed according to physical and chemical parameters. The study area, as well as sampling locations, have been shown in Fig. 1. In this study, 10 chemical parameters including calcium (Ca^2+^), sodium (Na^+^), potassium (K^+^), magnesium (Mg^+2^), bicarbonate (HCO_3_^−^), sulfate (SO_4_^2^^−^), chloride (Cl^−^), pH, TDS and electrical conductivity (EC) were used to evaluate the groundwater quality for drinking and agricultural purposes. Samples were collected in polyethylene bottles (1 L) and then the collected samples were kept in an ice box and then transferred to a fridge where they were stored at 4 °C until delivery to the laboratory. All water samples were analyzed according to the Standard Methods for Examination of Water and Wastewater and using titration method permanent hardness, magnesium and calcium were measured [14], [15], [16], [17], [18], [19], [20]. The concentration of hydrogen ion (pH) and electrical conductivity was also analyzed with pH meter (model wtw, Esimetrwb) and turbidity meter (model Hach 50161/co 150 model P2100Hach, USA), respectively [21], [22], [23], [24], [25], [26], [27], [28]. On the other hand, Values of, SO_4_^2^^−^ and Cl^−^ were obtained using spectrophotometer technique. In this study, various indices and ratios such as Sodium Absorption Ratio (SAR), Soluble Sodium Percentage (SSP), Residual Sodium Carbonate (RSC), Permeability Index (PI), Total Hardness (TH), Magnesium hazard (MH), Kelly׳s Ratio (KR), Pollution Index (PI), and Sodium percentage (Na %) were also determined that showed in Table 5. Then, to calculate WQI, the weight for physical and chemical parameters were determined with respect to the relative importance of the overall water quality for drinking water purposes.

**Fig. 2:**
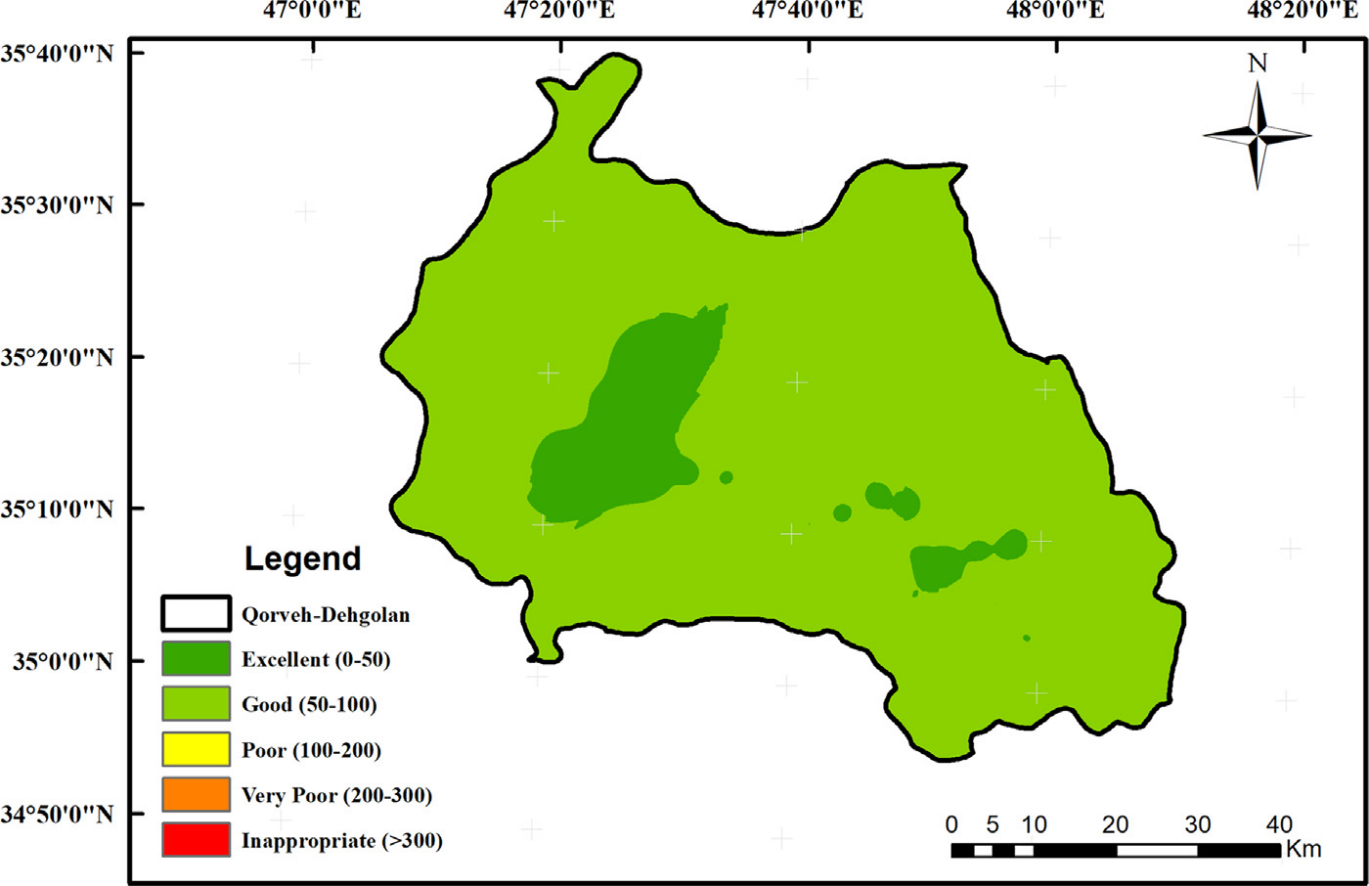
Spatial distribution map of water quality index.

All data of this study were statistically analyzed, and using a SPSS (IBM Corp. Released 2016. IBM SPSS Statistics for Windows, Version 24.0. Armonk, NY: IBM Corp), a correlation matrix was run. In order to describe groundwater quality and also possible pathways of geochemical changes, major ion chemical data have been drawn on Piper trilinear diagram (Piper 1944) in Fig.3. The distribution map of water quality index has been shown in Fig. 2

## 3 Drinking water quality index (DWQI)

The value of physio-chemical parameters has been determined to calculate the WQI formula. Also, it should be noted that assign of these parameters has been according to the relative importance of parameters in the overall quality of water for drinking objectives. The relative weight was calculated via the below equation [1].

(1) $$W_{i}=\sum \frac{W_{i}}{\sum_{i=1}^{n}W_{i}}$$

In this equation, the relative weight of each parameter is *W_i_*, and n refers to the number of parameters. Table 1 shows the weight (*w_i_*) and relative weight (*W_i_*) of each chemical parameter. For each parameter, the quality rating scale is calculated by dividing its concentration in each water sample to its respective standards (released by World Health Organization 2011) and finally multiplied the results by 100.

(2) $$q_{i}=\left(\frac{C_{i}}{S_{i}}\right)\times 100$$

where, *q_i_* shows the quality rating, *C_i_* refer the concentration of each chemical parameter in each sample (mg/L) and *S_i_* is the standard limit for each chemical parameter (mg/L) based on the guidelines of the WHO (2011). In the final of WQI calculating, the *SI_i_* was first assigned for each parameter and then the sum of *S_i_* values gave the water quality index for each sample [1].

(3) $$SI_{i}=W_{i}\times q_{i}$$

(4) $$WQI=\sum_{i=1}^{n}SI_{i}$$

where, *SI_i_* represents the sub-index of parameter, *q_i_* refers to the rating based on concentration of its parameter, and n is the number of parameters
